# Plant growth regulation of Bt-cotton through *Bacillus* species

**DOI:** 10.1007/s13205-013-0154-0

**Published:** 2013-07-20

**Authors:** Pavan Kumar Pindi, Tasleem Sultana, Praveen Kumar Vootla

**Affiliations:** Department of Microbiology, Palamuru University, Mahabubnagar, 509001 Andhra Pradesh India

**Keywords:** *Bacillus* sp. PU-7, Cotton seed varieties, IAA, Proline, Proteins, Sugars

## Abstract

Deccan plateau in India periodically experiences droughts due to irregular rain fall and the soil in many parts of the region is considered to be poor for farming. Plant growth promoting rhizobacteria are originally defined as root-colonizing bacteria, i.e., *Bacillus* that cause either plant growth promotion or biological control of plant diseases. The study aims at the isolation of novel *Bacillus* species and to assess the biotechnological potential of the novel species as a biofertilizer, with respect to their plant growth promoting properties as efficient phosphate-solubilizing bacteria. Seven different strains of *Bacillus* were isolated from cotton rhizosphere soil near boys’ hostel of Palamuru University which belongs to Deccan plateau. Among seven isolated strains, *Bacillus* strain-7 has shown maximum support for good growth of eight cotton cultivars. This bacterial species is named *Bacillus* sp. PU-7 based on the phenotypic and phylogenetic analysis. Among eight cotton cultivars, Mahyco has shown high levels of IAA, proteins, chlorophyll, sugars and low level of proline. Efficacy of novel *Bacillus* sp. PU-7 with Mahyco cultivar has been checked experimentally at field level in four different cotton grown agricultural soils. The strains supported plant growth in almost all the cases, especially in the deep black soil, with a clear evidence of maximum plant growth by increased levels of phytohormone production and biochemical analysis, followed by shallow black soil. Hence, it is inferred that the novel isolate can be used as bioinoculant in the cotton fields.

## Introduction

A group of rhizosphere bacteria (rhizobacteria) that exert a beneficial effect on plant growth is referred to as plant growth promoting rhizobacteria (PGPR), belongs to several genera: e.g., *Agrobacterium, Alcaligenes, Arthrobacter, Actinoplanes, Azotobacter*, *Bacillus*, *Pseudomonas* sp., *Rhizobium*. Multiple species of *Bacillus* and *Paenibacillus* are known to promote plant growth. The principal mechanisms of growth promotion include production of growth stimulating phytohormones, solubilization and mobilization of phosphate, siderophore production, antibiosis, i.e., production of antibiotics, inhibition of plant ethylene synthesis, and induction of plant systemic resistance to pathogens (Richardson et al. 2009; Idris et al. 2007; Gutierrez-Manero et al. 2001; Whipps 2001).

Ability of *Bacillus* sp. to produce organic acid such as gluconic, citric and fumaric acids under P-limiting conditions may increase the solubility of poorly soluble phosphorus. Several soil bacteria and fungi notably species of *Pseudomonas*, *Bacillus* and *Aspergillus*, etc., secrete organic acids and lower the pH in their vicinity to bring about solubilization of bound phosphates in soil (Sundra Rao and Sinha 1963). The N_2_-fixing bacterium associated with nonlegumes includes species of *Achromobacter*, *Alcaligenes*, *Arthrobacter*, *Acetobacter*, *Azomonas*, *Beijerinckia* and *Bacillus.* Among PGPR species, IAA producing bacteria are belonging to *Aeromonas* (Halda-Alija 2003), *Azotobacter* (Ahmad et al. 2008) and *Bacillus* (Swain et al. 2007).

Recently, Choudhary and Johri (2008) explicated the mechanisms and role of *Bacillus* species as inducers of systemic resistance in relation to plant–microbe interactions and demarcated the pathways involved in their regulation. Species of *Bacillus* are common inhabitants among the resident microflora of inner tissues of various species of plants, including cotton, grape, peas, spruce, and sweet corn, where they play an important role in plant protection and growth promotion (Berg et al. 2005; Shishido et al. 1999; Bell et al. 1995).

### Phytostimulation

Root-colonizing species of *Bacillus* and *Paenibacillus* are well known for the enhancement of plant growth (Idris et al. 2007). Growth promoting effects of various PGPRs are due to bacterial production of plant growth regulators such as indole-3-acetic acid (IAA), gibberellins, and cytokinins (Bottini et al. 2004; Bloemberg and Lugtenberg 2001). 80 % of rhizosphere colonizing bacteria has been reported positive for IAA production. Idris et al. (2004) showed production of substances with auxin (IAA)-like bioactivity from strains of *Bacillus subtilis/B. amyloliquefaciens* including strain FZB42. Gutierrez-Manero et al. (2001) has confirmed the production of gibberellins from *B. pumilus* and *B. licheniformis*. IAA plays an important key component in shaping plant root architecture such as root vascular tissue differentiation, regulation of lateral root initiation, polar root hair positioning, and root gravitropism (Aloni et al. 2006). Production of IAA from Gram-positive bacterium *B. amyloliquefaciens* FZB42 was first demonstrated by Idris et al. (2007) and its production was enhanced when the bacterium was fed with tryptophan. Application of phosphate solubilizers alone or in combination with nitrogen fixers has been found beneficial for cotton and wheat fields (Zaidi and Khan 2005; Kundu and Gaur 1980).

### Production of phytohormones by PGPR

The production of phytohormones by PGPR is the most important mechanisms by which many rhizobacteria promote plant growth (Spaepen et al. 2007). Phytohormones are signal molecules acting as chemical messengers and play a fundamental role as growth and development regulators in the plants. Phytohormones are organic compounds that in extremely low concentrations influence biochemical, physiological and morphological processes in plants, and their synthesis is finely regulated (Fuentes-Ramírez and Caballero-Mellado 2006). Numerous fungal and bacterial species can produce phytohormones (Tsavkelova et al. 2006). The phytohormone producing ability is widely distributed among bacteria associated with soil and plants. Studies have demonstrated that the PGPR can stimulate plant growth through the production of auxins (IAA) (Spaepen et al. 2008), gibberellins (Bottini et al. 2004) and cytokinins (Timmusk et al. 1999), or by regulating the high levels of endogenous ethylene in the plant (Glick et al. 1998).

### Indole acetic acid (IAA) producing rhizobacteria

Many important plant–microbial interactions center on the production of auxins, IAA being the main plant auxin. It is responsible for the division, expansion and differentiation of plant cells and tissues and stimulates root elongation. IAA synthesis has been detected in many rhizobacteria as well as in pathogenic, symbiotic and free living bacterial species (Costacurta and Vanderleyden 1995; Tsavkelova et al. 2006).

### Siderophore-producing rhizobacteria

Siderophores are produced by various types of bacteria in response to iron deficiency which occur in neutral to alkaline pH soils due to low iron solubility at elevated pH (Sharma and Johri 2003). Iron is essential for cellular growth and metabolism such that Fe acquisition through siderophore production plays an essential role in determining the competitive fitness of bacteria to colonize plant roots and to compete for iron with other microorganisms in the rhizosphere (Crowley and Gries 1994; Crowley 2006). Siderophore-producing organisms can prevent the proliferation of pathogenic microorganisms by sequestering Fe^3+^ in the area around the root (Siddiqui 2006).

## Materials and methods

The experiments were carried out in the Department of Microbiology, Palamuru University, Mahabubnagar, Andhra Pradesh, India.

### Sample collection

Cotton rhizosphere soil sample was collected near boys’ hostel of Palamuru University and screened for *Bacillus* species by serial dilution of the sample and dilution of 10^−4^ was spread on Soya bean trypticase agar.

### Preparation of standard inoculums of seven species of *Bacillus*

Inoculums of seven isolates of *Bacillus* sp. were prepared in selective medium. 150 ml broth medium was inoculated in 500-ml conical flask and incubated at 28 °C under shaking at 100–150 rpm for 3 days to give an optical density of 0.5. Broth culture of *Bacillus* sp. was inoculated in to peat (100 ml kg^−1^ of peat) which was sterilized at 121 °C and 15 psi pressure for 1 h. Peat-based *Bacillus* inoculums were incubated at 28 °C by adding 10 % sugar solution for 3–4 days to increase the population up to 10^8^ CFU ml^−1^. *Bacillus* inoculations having at 10^8^ MPN bacterial cells per gram of peat were applied to cotton seed as seed coating for a period of 90 days in sterilized soil.

### Preparation of sterilized soil

1:1 ratio of sand and red soil were mixed properly and sterilized in an autoclave at 121 °C, 15 lbs pressure.

### Genomic DNA extraction

The genomic DNA from the bacterial cells was obtained using a modification of the method described by Sambrook et al. (1989). The bacterial cells from pure culture were harvested by centrifugation (12,000 rpm) for 2 min, and the cell pellets were mixed with 600 μl of lysis buffer [10 mM Tris–HCl, 1 mM EDTA (pH 7.5), 0.5 % SDS, 100 g ml^−1^ proteinase C] and incubated at 37 °C for 1 h after the addition of 100 μl 5 M NaCl, and 80 μl CTAB/NaCl buffer. Samples were incubated at 65 °C for 10 min and samples were cooled to room temperature, followed by extraction of the aqueous phase with an equal volume of chloroform:isoamyl alcohol (24:11, v/v) and then with an equal volume of phenol:chloroform:isoamylalcohol (25:24:1, v/v) which was centrifuged at 12,000 rpm and 4 °C for 10 min. Isopropanol (0.6×) was mixed with the aqueous phase, and centrifuged at 12,000 rpm and 4 °C for 10 min. The DNA pellets were vacuum dried, and then dissolved in Tris buffer [10 mM Tris–HCl, and 1 mM EDTA (pH 7.5)].

### PCR analysis

The small subunit rRNA gene of each sample’s culture DNA was amplified using 16S rRNA Universal primers. The PCR amplification reaction mixture of 50 μl contained 4 μl bacterial DNA (nearly 200 ng), 1 μl Taq-DNA polymerase, 5 μl of Taq buffer, 5 μl of 2 mM dNTP mix, 5 μl of forward primer (10 pM μl^−1^) and 5 μl of reverse primer (10 pM μl^−1^). Amplification was carried out in a Bio-Rad thermo cycler run for 30 cycles. In each cycle denaturation was done at 94 °C for 20 s, annealing at 48 °C for 20 s and extension was done at 72 °C for 40 s and a final extension was carried out for 5 min at 72 °C at the end of all 30 cycles. The amplified DNA fragment of approximately 1,542 bp was separated on a 1 % agarose gel and purified by Qiagen spin columns (Mullis 1990; Barlett and Stirling 2003).

### 16S rRNA gene sequencing and phylogenetic tree construction

For 16S rRNA gene sequencing, DNA was prepared using the Mo Bio microbial DNA isolation kit (Mo Bio Laboratories Inc., Solano Beach, CA, USA) and sequenced as described previously (Lane 1991). The 1,502 nucleotides of 16S rRNA gene sequence of the isolate were subjected to BLAST sequence similarity search (Altschul et al. 1990) and EzTaxon (Chun et al. 2007) to identify the nearest taxa. All the 16S rRNA gene sequences belonging to the family “*Bacillaceae*” were downloaded from the database (http://www.ncbi.nlm.nih.gov), aligned using the CLUSTAL_X program (Thompson et al. 1997) and the alignment corrected manually. Phylogenetic trees were constructed using two tree-making algorithms, the maximum likelihood (ML) using the PhyML program (Guindon and Gascuel 2003) and Neighbor joining method (Saitou and Nei 1987) using the PHYLIP package, version 3.5 (Felsenstein 1993) and the resultant tree topologies were evaluated by bootstrap analysis based on 1,000 resamplings using the SEQBOOT and CONSENSE programs in the PHYLIP package. Pair-wise evolutionary distances were computed using DNADIST program with the Kimura 2-parameter model as developed by Kimura (1980).

### Phenotypic characterization of novel *Bacillus* sp. PU-7

Cell morphology and motility were studied using a light microscope. Motility was assessed on TSA medium containing l^−1^ pancreatic digest of casein (17 g), papaic digest of soyabean meat (3 g), sodium chloride (5 g), dipotassium hydrogen phosphate (2.5 g), dextrose (2.5 g) and agar (0.4 g). Growth at different temperatures, salt tolerance, biochemical characteristics, carbon assimilation, H_2_S production and the sensitivity of the culture to different antibiotics were determined by previously described methods (Lanyi 1987; Smibert and Krieg 1994). Biochemical characteristics were also double checked with Hi25™ Enterobacteriaceae identification kit (Cat #KB003) and HiCarbohydrate™ kit parts A, B and C (Cat #KB009 of HiMedia, Mumbai, India) according to the manufacturer’s protocol. Growth of PU1^T^ at different pHs was checked on NA medium buffered either with citric acid–NaOH (for pH 5 and 6), phosphate (for pH 7 and 8), glycine–NaOH (for pH 9 and 10) or Tris buffer (for pH 11 and 12).

### Production of IAA from *Bacillus* sp. PU-7

IAA production was detected as described by Brick et al. (1991). *Bacillus* culture was grown for 48 h on the respective media at 36 °C. Well-grown cultures were centrifuged at 3,000 rpm for 30 min. The supernatant (2 ml) was mixed with two drops of orthophosphoric acid and 4 ml of the Salkowski reagent (50 ml, 35 % of perchloric acid, 1 ml 0.5 M FeCl_3_ solution). Development of pink color indicates IAA production.

### Siderophore production from *Bacillus* sp. PU-7

Siderophore production was detected by Schwyn and Neilands (1987) using blue agar plates containing the dye chrome azurol S. An orange halo around the colony is indicative of siderophore production.

### Phosphate solubilization of *Bacillus* sp. PU-7

For phosphate solubilization assay, a medium containing 2 g yeast extract, 20 g glucose, 2 g tri calcium phosphate, 60 mg actidione, and 15 g agar mixed with 1,000 ml water, adjusted to pH 7, was used. A loopful inoculum of strain *Bacillus* sp. PU-7 was placed in the center of petri dishes containing the media and incubated at 28 °C for 5 days. Bacterial colony forming clear zone was considered as phosphate solubilizer (Rosas et al. 2006).

### Biochemical analysis of cotton plants inoculated with *Bacillus* sp. PU-7

#### Extraction and analysis of total protein

The total protein was extracted by homogenizing 0.5 g plant tissue in 10 ml of 0.2 M perchloric acid. The homogenate was centrifuged at 5,000*g* for 10 min at 24 °C. Ethanol–ether–chloroform (2:2:1; v/v/v) solvent mixture was used twice for the extraction of the pellet. To the residue, 0.2 M NaOH was added and left overnight. The supernatant was used for total protein estimation (Lowry et al. 1951).

#### Estimation of sugars

Total soluble sugar was analyzed by heating 1 g of the plant tissue with 0.2 % anthrone reagent and reading the intensity at 625 nm using UV–VIS spectrophotometer (Spectronic D20) (Mahadevan and Shridhar 1986).

#### Estimation of proline

Proline estimation was carried out as described by Bates et al. (1973). Fresh mass of 0.5 g leaf tissue was taken and homogenized in 5 ml of 3 % (w/v) sulphosalicylic acid. The residue was removed by centrifugation at 5,000 rpm for 10 min and the supernatant was filtered through Whatman # 2 filter paper. The filtrate was mixed with an equal volume of ninhydrin and glacial acetic acid and incubated at 95 °C for 1 h. The reaction was terminated by placing in an ice bath for about 30 min and then extracted with 4 ml toluene by mixing vigorously for 15 s. The toluene phase containing the chromophore was aspirated, warmed to room temperature for 10 min and the proline content was determined colorimetrically and expressed in mg g^−1^.

#### Estimation and extraction of chlorophyll

Chlorophyll pigment was extracted from 1 g of cotton leaves inoculated with *Bacillus* sp. PU-7 in 80 % acetone and estimated as described by Harborne (1973). The extracts were filtered in dark. OD values of filtrate were measured at 650 and 663 nm in UV–VIS spectrophotometer. The amount of total chlorophyll was calculated by Arnon’s formula.

#### Phytohormone production from cotton plants inoculated with *Bacillus* sp. PU-7

##### Estimation of IAA

One gram of leaf sample was crushed with 1 ml of phosphate buffer. Samples were centrifuged and two drops of perchloric acid were added to the supernatant to make up the volume to 2 ml with Salkowski reagent (i.e., 2 % 0.5 M FeCl_3_ in 35 % perchloric acid). OD values were taken after 25 min at 530 nm by UV–VIS spectrophotometer. Standard graph was prepared by plotting concentration of IAA in μg ml^−1^ vs optical density at 530 nm.

##### Collection of soil samples from different agricultural fields of cotton

Four different soil samples were collected from cotton fields of Mahabubnagar District, viz., shallow black soil from Malleboinpally, red soil from Makthal, deep black soil from Kalwakurthy and sandy soil from Narayanpet.

##### Physico-chemical characteristics of soil samples of agricultural fields of cotton

Soil available nitrogen was estimated by alkaline potassium permanganate method (Subbiah and Asija 1956), available phosphorous was determined by Bray and Kurtz (1945) and potassium determined by flame photometrically (Jackson 1973).

### Statistical analysis

A statistical analysis was performed using a statistical software SPSS for Windows version 17.0. ANOVA, *t* test was applied on subjects to know the significance of multiple mean differences and mean difference, respectively. *P* values are significant at 5 % level (or 95 confidence level).

## Results

### Isolation of novel PGPR *Bacillus* sp. PU-7

**Fig. 1:**
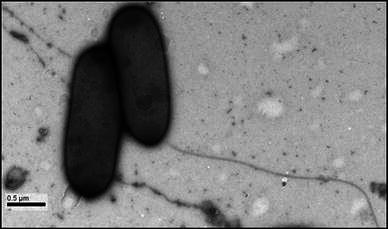
Electron micrograph of negatively stained cells of *Bacillus* sp. PU-7. *Bar* 0.5 μm

The main aim of present investigation is to isolate novel and efficient *Bacillus* species from cotton rhizosphere soil near boy’s hostel in Palamuru University. Seven species of *Bacillus* were isolated, among them *Bacillus* strain-7 has shown maximum growth of plant with 8 cultivars of cotton from 17 cultivars tested (Table 1). We subsequently named the isolated strain as *Bacillus* sp. PU-7 (Fig. 1) based on 16S rRNA gene sequence.

**Table 1:** Plant growth parameters of 17 cotton cultivars (60 days old) with 7 different isolates of *Bacillus*

Strain/seed variety	Control	Strain-1	Strain-2	Strain-3	Strain-4	Strain-5	Strain-6	Strain-7
Mahyco	+	++	+	++	+	+	+	+++
Ajeet	+	+	+	−	++	+	+	++
Rashi	+	−	++	+	−	++	−	+++
Tulasi	+	++	+	+	+	−	+	++
Marvel	+	+	−	−	++	−	+	+++
Bunni	+	+	++	−	−	+	++	++
PCH-125	+	++	+	+	++	+	−	++
Nusun	+	++	−	+	+	++	++	+++
Kaveri	+	−	++	+	+	+	+	+++
Raj seeds	+	+	+	−	+	+	+	+++
Super seeds	±	+	+	+	+	+	++	+
Veda	+	+	−	++	+	+	+	++
Brahmaputra	±	+	+	+	+	+	+	+
S99Bt	±	+	+	+	+	+	−	++
Obama Bt	+	+	+	−	+	−	+	+++
Bunni seeds	+	−	++	++	−	++	+	++
Sunny (NCS-108)	+	+	+	++	+	++	+	+++

+++ plant growth above 40 cm, ++ plant growth below 30 cm, + plant growth below 20 cm, ± plant growth below 15 cm, − plant growth below 10 cm

### Description of novel *Bacillus* sp. PU-7

**Fig. 2:**
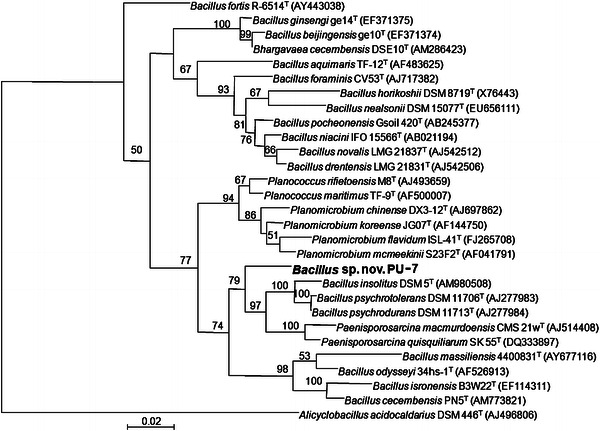
Phylogenetic tree based on 16S rRNA gene sequences showing the relationship of *Bacillus* sp. PU-7 with the species of the genus *Bacillus.* Phylogenetic tree was constructed using the maximum likelihood method. *Numbers at nodes* are bootstrap values. The *bar* represents 0.02 substitutions per alignment position

Cells are Gram-positive, motile with single mono-polar flagellum, rod-shaped (0.6–0.7 μm in width and 1.6–2 μm in length) and occur singly and multiply by binary fission. Colonies on nutrient agar are circular, 1–2 mm in diameter, smooth, cream in color, opaque, crateri form and entire. Cells grow from 18 to 40 °C with an optimum temperature of 37 °C and tolerate up to 9.0 % NaCl (w/v). Growth occurs in a pH range of 7–10, designated strain PU1^T^ was isolated from cotton rhizosphere soil near the boy’s hostel of Palamuru University, Mahabubnagar district, Andhra Pradesh, India. Cells of the strain PU1^T^ are positive for catalase, oxidase, phosphatase, lipase and urease and negative for gelatinase, amylase, protease, cellulase, lysine decarboxylase and ornithine decarboxylase (Table 2). The 16S rRNA gene sequence analysis indicated *Bacillus psychrodurans* and *Bacillus psychrotolerans* members of family “Bacillaceae” (phylum “Firmicutes”) are the closest related species with a sequence similarity of 96.0–96.2 %. Other members of the family “Bacillaceae” had sequence similarities of <96.0 %. Based on the above-mentioned phenotypic and phylogenetic characteristics, strain PU-7 is proposed as the representative of a new species, novel *Bacillus* sp. PU-7 (Fig. 2).

**Table 2:** Characteristics that differentiate the strain PU-7 with the closely related species of the genus *Bacillus*

Characteristic	PU-7	*Bacillus psychrodurans* DSM 11713^T^	*Bacillus psychrotolerans* DSM 11706^T^	*Bacillus insolitus* DSM 5^T^
Cell morphology	Rods	Rods	Regular rods	Rods
Cell size (μm)	0.6–0.7 × 1.6–2	0.5–0.6 × 2–5	0.4–1 × 2–7	1.0–1.5 × 1.6–2.7
Nitrate reduction	−	+	−	−
Salinity tolerance (%)	9	5	3	2
Temperature range (°C)	18–40	−2 to 30	−2 to 30	0–25
Anaerobic growth	−	+	−	−
β-Galactosidase	−	w	−	+
Lysine decarboxylase	−	w	w	+
Ornithine decarboxylase	−	+	+	+
Nitrate reduction	−	+	−	+
Hydrolysis of				
DNA	−	+	+	+
Gelatin	−	+	−	+
Starch	−	+	+	−
Tween 60	+	w	+	−
Urea	+	−	−	w
Acid production from				
d-Glucose,	+	+	w	−
l-Arabinose,	−	w	w	−
d-Xylose	+	w	w	−
d-Mannitol	+	+	w	−
Utilization of				
Citrate	+	−	−	+

+ positive, − negative, *w* weak

**Fig. 3:**
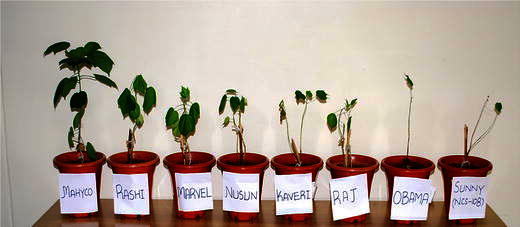
Eight different cultivars of cotton inoculated with novel *Bacillus* sp. PU-7

*Bacillus* sp. PU-7 was tested for plant growth promoting characteristics (Table 3). Eight cultivars which have shown maximum growth with *Bacillus* sp. PU-7 (Fig. 3) were analyzed for biochemical characters and phytohormone production. Among eight cultivars of cotton, Mahyco has shown maximum levels of IAA, proteins sugars, chlorophyll, and minimum content of proline and Sunny (NCS-108) has shown minimum levels of IAA, proteins, sugars chlorophyll and high level of proline (Table 4).

**Table 3:** Plant growth promoting characteristics of novel *Bacillus* sp. PU-7

Characteristics	*Bacillus* sp. PU-7
Phosphate solubilization test	+
IAA production	+
Siderophore production	+

**Table 4:** Phytohormone and biochemical production of 8 cotton cultivars (60 days old) inoculated with *Bacillus* sp. PU-7 (mean ± SE)

Cultivar	IAA (μg g^−1^)	Proteins (mg g^−1^)	Sugars (mg g^−1^)	Chlorophyll (mg g^−1^)	Proline (μg g^−1^)
Mahyco	749.67 ± 22.81	270 ± 11.55	129 ± 5.86	156.67 ± 6.67	340 ± 30.55
Rashi	657 ± 23.16	218.34 ± 15.90	140.66 ± 15.45	134 ± 8.72	350.67 ± 25.57
Marvel	658.34 ± 13.64	223 ± 4.62	118 ± 8.39	136.67 ± 14.53	330.00 ± 25.17
Nusun	634 ± 20.23	208 ± 15.62	122.67 ± 11.85	113.33 ± 6.67	325.33 ± 16.38
Kaveri	561 ± 10.69	175 ± 8.66	101.67 ± 10.14	113.33 ± 8.82	343.34 ± 29.06
Raj seeds	536.34 ± 14.84	165 ± 10.41	129 ± 12.42	89.67 ± 5.78	258.33 ± 13.64
Obama	502 ± 13.00	140 ± 2.31	126.67 ± 17.64	106.67 ± 14.53	240 ± 15.28
Sunny	431 ± 20.60	124 ± 3.06	103.33 ± 6.67	156.67 ± 8.82	277 ± 11.36
*P* value	0.0001**	0.0001**	0.24	0.05*	0.05*

* Significant; ** Highly significant

### Efficacy of novel *Bacillus* sp. PU-7 as a PGPR

Based on the above discussed results, it was apparent that *Bacillus* sp. PU-7 could be as used as a PGPR in bio-formulation of poor soils that support least plant growth. We have collected four soil samples that were known to be poor for plant growth as described in “Materials and methods” and NPK and trace elements were determined (Table 5). These soil samples were inoculated with *Bacillus* sp. PU-7 and uninoculated sample was taken as control. Mahyco cultivar was grown in these 4 different soils for a period of 90 days in order to see the efficiency of novel isolate in natural conditions. Mahyco growth (physical growth parameters, phytohormone production and biochemical characteristics) was good in all soil types, but maximum growth was recorded in deep black soil followed by shallow black soil and minimum growth was observed in sandy soil followed by red soil (Tables 6, 7).

**Table 5:** Physico-chemical characteristics and available sulphur and micronutrients content of four agricultural soils of Mahabubnagar dist

GPS location	Soil type	pH	N	P	K	S	Fe	Mn	Zn	Cu
Malleboinpally lati 78.13388, long 16.77465	Shallow black soil	8.0	220.10	95.62	118.42	8.8	2.27	24.15	0.26	0.25
Makthal lati 77.527836, long 16.724264	Red soil	7.0	199.47	86.19	103.69	11.2	5.32	26.31	0.98	0.24
Kalwakurthy lati 78.492231, long 16.66739	Deep black	8.0	248.95	103.26	132.14	8.2	2.24	32.123	0.35	0.51
Narayanpet lati 77.49425, long 16.74231	Sandy soil	8.0	192.96	79.87	98.78	3.2	0.13	9.48	0.05	0.03

NPK in kg ha^−1^, sulphur and micronutrients in ppm

**Table 6:** Plant growth parameters, phytohormone production and biochemical characteristics of Mahyco cotton cultivar in four different agricultural soils of Mahabubnagar district

Soil type	Location	Combination	Height of the plant (cm)		Plant fresh weight (g)		Plant dry weight (g)	
			*S*	*R*	*S*	*R*	*S*	*R*
Shallow black soil	Malleboinpally	Control	22.4 ± 0.00	11.3 ± 0.00	3.9 ± 0.00	1.45 ± 0.00	0.52 ± 0.00	0.4 ± 0.00
		M + BP	33.2 ± 0.52*	22.3 ± 0.34*	4.52 ± 0.014*	1.74 ± 0.04*	0.67 ± 0.028*	0.56 ± 0.014*
Red soil	Makthal	Control	20.2 ± 0.00	11.0 ± 0.00	3.85 ± 0.00	1.4 ± 0.00	0.5 ± 0.00	0.39 ± 0.00
		M + BP	30.7 ± 0.17*	20.3 ± 0.14*	4.24 ± 0.02*	1.66 ± 0.014*	0.61 ± 0.01*	0.61 ± 0.00*
Deep black soil	Kalwakurthy	Control	25.4 ± 0.00	12.7 ± 0.00	3.99 ± 0.00	1.89 ± 0.00	0.58 ± 0.00	0.48 ± 0.00
		M + BP	37.9 ± 0.02*	23.4 ± 0.14*	4.62 ± 0.014*	1.95 ± 0.025*	0.67 ± 0.02*	37.9 ± 0.02*
Sandy soil	Narayanpet	Control	20.1 ± 0.00	10.2 ± 0.00	3.45 ± 0.00	1.34 ± 0.00	0.45 ± 0.00	0.32 ± 0.00
		M + BP	30.16 ± 0.013*	20.16 ± 0.14*	4.20 ± 0.00*	1.62 ± 0.007*	0.61 ± 0.007*	0.51 ± 0.007*

*M* Mahyco cotton cultivar, *BPBacillus* sp. PU-7, *S* shoot, *R* root

* Significant at 0.05

**Table 7:** Phytohormone production and biochemical characteristics of Mahyco cotton cultivar in four different agricultural soils (with mean ± SE) of Mahabubnagar district

Soil type	Location	Combination	IAA (μg g^−1^)	Proteins (mg g^−1^)	Sugars (mg g^−1^)	Chlorophyll (mg g^−1^)	Proline (μg g^−1^)
Shallow black soil	Malleboinpally	Control	382 ± 0.00	62 ± 0.00	77 ± 0.00	62.9 ± 0.00	520 ± 0.00
		M + BP	782.67 ± 4.24*	175.67 ± 1.41*	132.33 ± 1.41*	147.67 ± 2.12*	408.33 ± 10.21*
Red soil	Makthal	Control	650 ± 0.00	102 ± 0.00	92.00 ± 0.00	109 ± 0.00	680 ± 0.00
		M + BP	715.33 ± 14.14*	137.67 ± 0.71*	109.00 ± 1.41*	113.00 ± 1.41*	556.67 ± 15.28*
Deep black soil	Kalwakurthy	Control	700 ± 0.00	69.00 ± 0.00	87.00 ± 0.00	70.1 ± 0.00	410 ± 0.00
		M + BP	811.67 ± 10.61*	219.00 ± 1.41*	151.00 ± 1.42*	182.33 ± 3.54*	316.67 ± 5.77*
Sandy soil	Narayanpet	Control	350 ± 0.00	58.00 ± 0.00	68.00 ± 0.00	52.3 ± 0.00	740 ± 0.00
		M + BP	635.67 ± 5.66*	99.00 ± 3.54*	99.00 ± 4.95*	107.33 ± 3.54*	646.67 ± 30.33*

* Significant at 0.05

## Discussion

Rhizosphere is defined as the soil influenced by roots, bacterial species that carry out functions which promote growth of plants. These bacteria are designated as PGPR (Martínez-Viveros et al. 2010). *Pseudomonas* and *Bacillus* genera are the most commonly investigated PGPR, and often the dominating bacterial groups in the rhizosphere (Morgan et al. 2005). *Bacillus* species have been reported to promote the growth of a wide range of plants (De Freitas et al. 1997; Kokalis-Burelle et al. 2002). Trials with rhizosphere-associated plant growth promoting N_2_-fixing and phosphate solubilising *Bacillus* species indicated yield increase in sorghum (Broadbent et al. 1977), maize (Pal 1998), rice (Sudha et al. 1999), sugar beet (Cakmakci et al. 1999), barley (Sahin et al. 2004) and apples (Aslantas et al. 2007).

McSpadden Gardener (2004) and Ona et al. (2003) demonstrated the enhancement of plant growth by *Bacillus* and *Paenibacillus*. They promote plant growth by the solubilization of phosphorus and production of phytohormones, such as IAA (Lal and Tabacchioni 2009). Among PGPR species, *Azospirillum* is one of the best studied IAA producers (Dobbelaere et al. 1999). Other IAA producing bacteria belonging to *Aeromonas* (Halda-Alija 2003), *Azotobacter* (Ahmad et al. 2008), *Bacillus* (Swain et al. 2007), *Burkholderia* (Halda-Alija 2003), *Enterobacter* (Shoebitz et al. 2009), *Pseudomonas* (Hariprasad and Niranjana 2009) and *Rhizobium* (Ghosh et al. 2008) genera have been isolated from different rhizosphere soils. Inoculation with IAA producing PGPR has been used to stimulate seed germination, to accelerate root growth and modify the architecture of the root system, and to increase the root biomass. In recent studies, Tsavkelova et al. (2007) have extended beyond individual strains as inoculants and reported an increase in the germination of orchid seeds (*Dendrobium moschatum*) inoculated with *Sphingomonas* sp. and IAA producing *Mycobacterium* sp. In addition to stimulating root growth, IAA producing bacteria can also be used to stimulate tuber growth. Swain et al. (2007) reported a positive effect of *B. subtilis* IAA producing strains on the edible tubercle *Dioscorea rotundata* L. in one of their studies. They applied a suspension of *B. subtilis* on the surface of the plants, which resulted in an increase in stem and root length, increased fresh weight of the stem and root, an increase in the root:stem ratio and increased numbers of sprouts as compared with non-inoculated plants. Phytohormones such as IAA may indirectly improve P acquisition by plants by increasing root growth (Marschner et al. 2011). *Bacillus* and *Paenibacillus* are also able to produce endospores, which enhances their persistence and viability in soils (Lal and Tabacchioni 2009; Nicholson 2002). Yao et al. (2006) described the cotton plant crop response to *B. subtilis* FZB 24 experimental conditions which has increased the 31 % average yield (t ha^−1^), 19 % bolls/plant mean number and 11 % mean plant height in cm.

In the present investigation *Bacillus* sp. PU-7 which was identified based on 16S rRNA gene sequence has shown positive results for plant growth promoting characteristics such as phosphate solubilization, IAA and siderophore production.

The ability of PGPRs to convert insoluble phosphorus (P) to an accessible form is an important trait for increasing the plant yields. The fact that certain microbes are capable of dissolving relatively insoluble phosphatic compounds has opened the possibility of inducing microbial solubilization of the phosphates in the soil (Zaidi et al. 2009). Rhizobacteria solubilize the mineral P in the rhizosphere and hence, provided soluble P to the plants. The cause of the mineral P solubilization could probably be due to secretion of organic acids such as gluconic, 2-ketogluconic, oxalic, citric, acetic, malic, and succinic acid, etc. It is clear from the present results that *Bacillus* sp. PU-7 is a potential phosphate solubilizer.

Bacilli strains that can solubilizes P and produce siderophore and IAA have widely been reported (Raddadi et al. 2008; Trivedi and Pandey 2008) and have been shown to promote the growth of maize and wheat (Beneduzi et al. 2008; Trivedi and Pandey 2008).

Efficient and potential isolate *Bacillus* sp. PU-7 has shown maximum growth and high levels of IAA, proteins, sugars, chlorophyll and proline with Mahyco cultivar. This novel isolate has shown tremendous growth in different agricultural soil types, but maximum growth was observed in black soil in terms of plant growth, phytohormonal production and biochemical characteristics.

## Conclusion

Novel *Bacillus* sp. PU-7 is an efficient isolate which is enhancing good growth of cotton plants in terms of physical growth parameters, phytohormonal, biochemical properties and supports plant growth in deep black soils followed by shallow black soils. Hence, this novel species with the biotechnological potential as a biofertilizer, with respect to their plant growth promoting properties can be used as bioinoculant in the cotton fields. It is concluded from present findings that efficient novel isolate can be used as a bioinoculant/PSB in different agricultural soils of cotton.

## References

[CR1] Ahmad F, Ahmad I, Khan MS (2008). Screening of free-living rhizospheric bacteria for their multiple plant growth promoting activities. Microbiol Res.

[CR2] Aloni R (2006). Role of cytokinin and auxin in shaping root architecture: regulating vascular differentiation, lateral root initiation, root apical dominance and root gravitropism. Ann Bot.

[CR3] Altschul S, Gish W, Myers EW, Lipman DJ (1990). Basic local alignment search tool. Mol Biol.

[CR4] Aslantas R, Cakmakci R, Sahin F (2007). Effect of plant growth promoting rhizobacteria on young apple trees growth and fruit yield under orchard conditions. Sci Hortic.

[CR5] Barlett JM, Stirling D (2003). A short history of the polymerase chain reaction. Methods Mol Biol.

[CR6] Bates LE, Waldren RP, Teare FD (1973). Rapid determination of free proline for water stress studies. Plant Soil.

[CR7] Bell CR (1995). Endophytic bacteria in grapevine. Can J Microbiol.

[CR8] Beneduzi A, Peres D, Da Costa PB, Bodanese Zanettini MH, Passaglia LM (2008). Genetic and phenotypic diversity of plant-growth-promoting bacilli isolated from wheat fields in southern Brazil. Res Microbiol.

[CR9] Berg G (2005). Impact of plant species and site on rhizosphere-associated fungi antagonistic to *Verticillium dahliae* Kleb. Appl Environ Microbiol.

[CR10] Bloemberg GV, Lugtenberg BFJ (2001). Molecular basis of plant growth promotion and biocontrol by rhizobacteria. Curr Opin Plant Biol.

[CR11] Bottini R, Cassan F, Picolli P (2004). Gibberellin production by bacteria and its involvement in plant growth promotion. Appl Microbiol Biotechnol.

[CR12] Bray R, Kurtz LT (1945). Determination of total organic and available forms of phosphorus in soils. Soil Sci.

[CR13] Brick JM, Bostock RM, Silverstone SE (1991). Rapid in situ assay for indole acetic acid production by bacteria immobilized on nitrocellulose membrane. Appl Environ Microbiol.

[CR14] Broadbent P, Bake KF, Franks N, Holland J (1977). Effect of *Bacillus* sp. on increased growth of seedlings in steamed and in non treated soil. Phytopathology.

[CR15] Cakmakci R, Kantar F, Algur OF (1999). Sugar beet and barley yields in relation to *Bacillus polymyxa* and *Bacillus megaterium* var. *phosphaticum* inoculation. J Plant Nutr Soil Sci.

[CR16] Choudhary DK, Johri BN (2008). Interactions of *Bacillus* sp. and plants-with special reference to induced systemic resistance (ISR). Microbial Res.

[CR17] Chun J (2007). Ez Taxon: a web based tool for the identification of prokaryotes based on 16S ribosomal RNA gene sequences. Int J Syst Evol Microbiol.

[CR18] Costacurta A, Vanderleyden J (1995). Synthesis of phytohormones by plant-associated bacteria. Crit Rev Microbiol.

[CR19] Crowley DE, Barton LL, Abadía J (2006). Microbial siderophores in the plant rhizosphere. Iron nutrition in plants and rhizospheric microorganisms.

[CR20] Crowley DE, Gries D, Manthey JA, Crowley DE, Luster DG (1994). Modeling of iron availability in the plant rhizosphere. Biochemistry of metal micronutrients in the rhizosphere.

[CR21] De Freitas JR, Banerjee MR, Germida JJ (1997). Phosphate-solubilizing rhizobacteria enhance the growth and yield but not phosphorus uptake of canola (*Brassica napus* L.). Biol Fertil Soil.

[CR22] Dobbelaere S, Croonenborghs A, Thys A, Vande Broek A, Vanderleyden J (1999). Phytostimulatory effect of *Azospirillum brasilense* wild type and mutant strains altered in IAA production on wheat. Plant Soil.

[CR23] Felsenstein J (1993) PHYLIP (phylogeny inference package), version 3.5.1. Department of Genome Sciences, University of Washington, Seattle

[CR24] Fuentes-Ramírez LE, Caballero-Mellado J, Siddiqui ZA (2006). Bacterial biofertilizers. PGPR: biocontrol and biofertilization.

[CR25] Ghosh S, Sengupta C, Maiti TK, Basu PS (2008). Production of 3-indolylacetic acid in root nodules and culture by a *Rhizobium* species isolated from root nodules of the leguminous pulse *Phaseolus mungo*. Folia Microbiol.

[CR26] Glick BR, Penrose DM, Li J (1998). A model for the lowering of plant ethylene concentrations by plant growth-promoting bacteria. J Theor Biol.

[CR27] Guindon S, Gascuel O (2003). A simple, fast, and accurate algorithm to estimate large phylogenies by maximum likelihood. Syst Biol.

[CR28] Gutierrez-Manero FJ, Ramos B, Probanza A, Mehouachi J, Talon M (2001). The plant growth promoting rhizobacteria *Bacillus pumilus* and *Bacillus licheniformis* produce high amounts of physiologically active gibberellins. Physiol Plant.

[CR29] Halda-Alija L (2003). Identification of indole-3-acetic acid producing freshwater wetland rhizosphere bacteria associated with *Juncus effusus* L. Can J Microbiol.

[CR30] Harborne JB (1973). Phytochemical methods.

[CR31] Hariprasad P, Niranjana SR (2009). Isolation and characterization of phosphate solubilizing rhizobacteria to improve plant health of tomato. Plant Soil.

[CR32] Idris EES (2004). Use of *Bacillus subtilis* as biocontrol agent. 6. Phytohormone action of culture filtrate prepared from plant growth promoting *Bacillus amyloliquefaciens* FZB24, FZB42, FZB45 and *Bacillus subtilis* FZB37. J Plant Dis Prot.

[CR33] Idris EES, Iglesias DJ, Talon M, Borriss R (2007). Tryptophan-dependent production of indole-3-acetic acid (IAA) affects level of plant growth promotion by *Bacillus**amyloliquefaciens* FZB42. Mol Plant Microbe Interact.

[CR34] Jackson ML (1973). Soil chemical analysis.

[CR35] Kimura M (1980). A simple method for estimating evolutionary rates of base substitutions through comparative studies of nucleotide sequences. J Mol Evol.

[CR36] Kokalis-Burelle N, Vavrina CS, Rosskopf EN, Shelby RA (2002). Field evaluation of plant growth promoting rhizobacteria amended transplant mixes and soil solarization for tomato and pepper production in Florida. Plant Soil.

[CR37] Kundu BS, Gaur AC (1980). Effect of nitrogen fixing and phosphate solubilizing microorganisms as single and composite inoculants on cotton. Ind J Mirobiol.

[CR38] Lal S, Tabacchioni S (2009). Ecology and biotechnological potential of *Paenibacillus polymyxa*: a minireview. Ind J Mirobiol.

[CR39] Lane DJ, Stackebrandt E, Goodfellow M (1991). 16S/23S rRNA sequencing. Nucleic acid techniques in bacterial systematics.

[CR40] Lanyi B (1987). Classical and rapid identification methods for medically important bacteria. Methods Microbiol.

[CR41] Lowry OH, Rosebrough NJ, Farr AL, Randall RJ (1951). Protein measurement with the Folin-phenol reagent. J Biol Chem.

[CR42] Mahadevan A, Shridhar R (1986). Methods in physiological plant pathology.

[CR43] Marschner P, Crowley D, Rengel Z (2011). Rhizosphere interaction between microorganisms and plants govern iron and phosphorus acquisition along the root axis—model and research methods. Soil Biol Biochem.

[CR44] Martínez-Viveros O, Jorquera MA, Crowley DE, Gajardo G, Mora ML (2010). Mechanisms and practical considerations involved in plant growth promotion by rhizobacteria. J Soil Sci Plant Nutr.

[CR45] McSpadden Gardener BB (2004). Ecology of *Bacillus* and *Paenibacillus* spp. in agricultural systems. Phytopathology.

[CR46] Morgan JA, Bending GD, White PJ (2005). Biological costs and benefits to plant–microbe interactions in the rhizosphere. J Exp Bot.

[CR47] Mullis KB (1990). The unusual origin of the polymerase chain reaction. Sci Am.

[CR48] Nicholson W (2002). Roles of *Bacillus* endospores in the environment. Cell Mol Life Sci.

[CR49] Ona O, Smets I, Gysegom P, Bernaerts K, Van Impre J, Prinsen E, Vanderleyden J (2003). The effect of pH on indole-3-acetic acid (IAA) biosynthesis of *Azospirillum brasilense* Sp7. Symbiosis.

[CR50] Pal SS (1998). Interaction of an acid tolerant strain of phosphate solubilizing bacteria with a few acid tolerant crops. Plant Soil.

[CR51] Raddadi N, Cherif A, Boudabous A, Daffonchio D (2008). Screening of plant growth promoting traits of *Bacillus thuringiensis*. Ann Microbiol.

[CR52] Richardson AE, Barea JM, McNeill AM, Prigent-Combaret C (2009). Acquisition of phosphorus and nitrogen in the rhizosphere and plant growth promotion by microorganisms. Plant Soil.

[CR53] Rosas SB, Andres JA, Rovera M, Correa NS (2006). Phosphate solubilizing *Pseudomonas putida* can influence rhizobia-legume symbiosis. Soil Biol Biochem.

[CR54] Sahin F, Cakmakci R, Kantar F (2004). Sugar beet and barley yields in relation to inoculation with N2-fixing and phosphate solubilizing bacteria. Plant Soil.

[CR55] Saitou N, Nei M (1987). The neighbor-joining method: a new method for reconstructing phylogenetic trees. Mol Biol Evol.

[CR56] Sambrook J, Fritschim EF, Maniatis T (1989). Molecular cloning: a laboratory manual.

[CR57] Schwyn B, Neilands JB (1987). Universal chemical assay for detection and determination of siderophore. Anal Biochem.

[CR58] Sharma A, Johri BN (2003). Growth promoting influence of siderophore-producing *Pseudomonas* strains GRP3A and PRS9 in maize (*Zea mays* L.) under iron limiting conditions. Microbiol Res.

[CR59] Shishido M, Breuil C, Chanway CP (1999). Endophytic colonization of spruce by plant growth promoting rhizobacteria. FEMS Microbiol Ecol.

[CR60] Shoebitz M, Ribaudo CM, Pardo MA, Cantore ML, Ciampi L, Curá JA (2009). Plant growth promoting properties of a strain of *Enterobacter ludwigii* isolated from *Lolium perenne* rhizosphere. Soil Biol Biochem.

[CR61] Siddiqui ZA, Siddiqui ZA (2006). PGPR: prospective biocontrol agents of plant pathogens. PGPR: biocontrol and biocontrol.

[CR62] Smibert RM, Krieg NR, Gerhardt P, Murray RGE, Wood WA, Krieg NR (1994). Phenotypic characterization. Methods for general and molecular bacteriology.

[CR63] Spaepen S, Vanderleyden J, Remans R (2007). Indole-3-acetic acid in microbial and microorganism-plant signaling. FEMS Microbiol Rev.

[CR64] Spaepen S, Dobbelaere S, Croonenborghs A, Vanderleyden J (2008). Effects of *Azospirillum brasilense* indole-3-acetic acid production on inoculated wheat plants. Plant Soil.

[CR65] Subbiah BV, Asija GL (1956). A rapid procedure for the estimation of available nitrogen in soils. Curr Sci.

[CR66] Sudha SN, Jayakumar R, Sekar V (1999). Introduction and expression of the cry1Ac gene of *Bacillus thuringiensis* in a cereal associated bacterium, *Bacillus polymyxa*. Curr Microbiol.

[CR67] Sundra Rao WVB, Sinha MK (1963). Phosphate dissolving organisms in soil and rhizosphere. Indian J Agric Sci.

[CR68] Swain MR, Naskar SK, Ray RC (2007). Indole-3-acetic acid production and effect on sprouting of Yam (*Dioscorea rotundata* L.) minisetts by *Bacillus subtilis* isolated from culturable cowdung microflora. Pol J Microbiol.

[CR69] Thompson JD (1997). The CLUSTAL_X windows interface: flexible strategies for multiple sequence alignment aided by quality analysis tools. Nucl Acids Res.

[CR70] Timmusk S, Nicander B, Granhall U, Tillberg E (1999). Cytokinin production by *Paenibacillus polymyxa*. Soil Biol Biochem.

[CR71] Trivedi P, Pandey A (2008). Plant growth promotion abilities and formulation of *Bacillus megaterium* strain B 388 (MTCC6521) isolated from a temperate Himalayan location. Indian J Microbiol.

[CR72] Tsavkelova EA, Klimova SY, Cherdyntseva TA, Netrusov AI (2006). Microbial producers of plant growth stimulators and their practical use: a review. Appl Biochem Micro.

[CR73] Tsavkelova EA, Cherdyntseva TA, Klimova SY, Shestakov AI, Botina SG, Netrusov AI (2007). Orchid-associated bacteria produce indole-3-acetic acid, promote seed germination, and increase their microbial yield in response to exogenous auxin. Arch Microbiol.

[CR74] Whipps JM (2001). Microbial interactions and biocontrol in the rhizosphere. J Exp Bot.

[CR75] Yao AV, Bochow H, Karimov S, boturov U, Sanginboy S, Sharipov K (2006) Effect of FZB24 *Bacillus subtilis* as a biofertilizer on cotton yields in field tests. Arch Phytopathol Plant Prot 39:1–6

[CR76] Zaidi A, Khan S (2005). Interactive effect of rhizospheric microorganisms on growth, yield and nutrient uptake of wheat. J Plant Nutr.

[CR77] Zaidi A, Khan MS, Ahemad M, Oves M (2009). Plant growth promotion by phosphate solubilizing bacteria. Acta Microbiol Immunol Hung.

